# Allele Specific Locked Nucleic Acid Quantitative PCR (ASLNAqPCR): An Accurate and Cost-Effective Assay to Diagnose and Quantify *KRAS* and *BRAF* Mutation

**DOI:** 10.1371/journal.pone.0036084

**Published:** 2012-04-30

**Authors:** Luca Morandi, Dario de Biase, Michela Visani, Valentina Cesari, Giovanna De Maglio, Stefano Pizzolitto, Annalisa Pession, Giovanni Tallini

**Affiliations:** 1 Dipartimento di Ematologia e Scienze Oncologiche “L e A Seragnoli”, Sezione di Anatomia Istologia e Citologia Patologica "M. Malpighi" Università di Bologna-AUSL Ospedale Bellaria, Bologna, Italy; 2 Dipartimento di Patologia Sperimentale Università di Bologna, Bologna, Italy; 3 SOC Anatomia Patologica Azienda Ospedaliero-Universitaria Santa Maria della Misericordia, Udine, Italy; Ospedale Pediatrico Bambino Gesu', Italy

## Abstract

The use of tyrosine kinase inhibitors (TKIs) requires the testing for hot spot mutations of the molecular effectors downstream the membrane-bound tyrosine kinases since their wild type status is expected for response to TKI therapy. We report a novel assay that we have called Allele Specific Locked Nucleic Acid quantitative PCR (ASLNAqPCR). The assay uses LNA-modified allele specific primers and LNA-modified beacon probes to increase sensitivity, specificity and to accurately quantify mutations. We designed primers specific for codon 12/13 *KRAS* mutations and *BRAF* V600E, and validated the assay with 300 routine samples from a variety of sources, including cytology specimens. All were analyzed by ASLNAqPCR and Sanger sequencing. Discordant cases were pyrosequenced. ASLNAqPCR correctly identified *BRAF* and *KRAS* mutations in all discordant cases and all had a mutated/wild type DNA ratio below the analytical sensitivity of the Sanger method. ASLNAqPCR was 100% specific with greater accuracy, positive and negative predictive values compared with Sanger sequencing. The analytical sensitivity of ASLNAqPCR is 0.1%, allowing quantification of mutated DNA in small neoplastic cell clones. ASLNAqPCR can be performed in any laboratory with real-time PCR equipment, is very cost-effective and can easily be adapted to detect hot spot mutations in other oncogenes.

## Introduction

Molecular therapy targeting transmembrane receptor tyrosine kinases with a variety of tyrosine kinase inhibitors has become part of the standard treatment for many patients with common forms of cancer. Evidence of both tyrosine kinase activation and lack of activating mutations in the tyrosine kinase downstream effectors is expected as a general precondition for successful patient treatment [1]. Among transmembrane tyrosine kinases the EGF receptor (EGFR) is one of the main therapeutic targets since it is active in both colorectal (CRC) and non-small cell lung cancers (NSCLC). The MAP kinase (MAPK) signaling cascade is a mainstream pathway that modulates many cell functions (e.g. proliferation, differentiation, apoptosis) following the activation of tyrosine kinase receptors like EGFR. *KRAS* and *BRAF* are key members of this pathway, constitutively active due to oncogenic mutations in ∼40% of human cancers, with a prevalence of mutation that varies considerably among tumors originating from different tissues [2]. *KRAS* oncogenic activation, largely due to codon 12–13 mutations, occurs in ∼40% of CRC [3], [4] and in ∼15% of NSCLC [5]. As expected, *KRAS* mutations have been associated with poor response to anti-EGFR therapy in patients with both CRC [3] and NSCLC [6]. Wild type *KRAS* is now considered a pre-condition to treat CRC patients with EGFR inhibitors like Cetuximab or Panitumumab [7], [8]. Oncogenic *BRAF* mutations occur in up to 15% of all human tumors, the vast majority (>90%) being c.1799:T>A substitutions that lead to the replacement of valine with aspartic acid (V600E) causing constitutive *BRAF* activation [9]. Melanoma (40–60%) [10] and papillary thyroid carcinoma (PTC; 40%–80%) [11] are the tumors with the highest incidence of *BRAF* mutations. While *BRAF* mutations are uncommon in NSCLC [12], they occur in ∼10–15% of CRC and are strongly associated with non-Lynch microsatellite unstable tumors and with the CpG island methylator phenotype [13]. Similar to *KRAS*, *BRAF* mutation has been correlated with lack of response to EGFR inhibitors in patients with advanced CRC and the impact of *BRAF* mutations on TKI treatment response is currently being investigated [14]. Furthermore, novel *BRAF* inhibitor molecules like vemurafenib are proving highly effective to treat patients with *BRAF* mutated tumors, like melanoma [15].

Additional reasons for molecular testing are the diagnostic or prognostic information that can be obtained by the analysis of tumors with a high prevalence of specific mutations as is the case for *KRAS* and pancreatic lesions [16] or *BRAF* and thyroid nodules [11].

The above considerations point to the necessity to test for *KRAS* and *BRAF* mutations. In fact, the advent of targeted therapy mandates the analysis of large numbers of tumors and is forcing the integration of molecular data into the routine workflow of cancer patients [1]. This can prove a challenge and underlines the importance of utilizing detection methods that are sensitive, rapid, reproducible and cost-effective.

Sanger sequencing is highly reliable and currently considered the “gold standard” technique for mutation detection [7], [8]. However, when applied to routine diagnostic use suffers from several limitations. Sanger sequencing is low throughput, requires several distinct steps (e.g. PCR, amplicon purification, labelling) each of which is exposed to contamination risk, is relatively dependent on the quality and integrity of DNA, and has a low analytical sensitivity, requiring at least 25% of mutated DNA-corresponding to at least 50% of neoplastic cells with an heterozygous mutated allele. Considering that many routine samples contain large numbers of non-neoplastic reactive/inflammatory cells, dissection of specimens prior to DNA extraction is usually necessary to enrich for neoplastic cells and to avoid false negative results. A variety of more sensitive methods based on different approaches are utilized to overcome the limitations of Sanger sequencing, but many of them can be time-consuming, labor-intensive, expensive or require the use of sophisticated platforms not always affordable by pathology laboratories [17], [18].

We here describe a new assay that we have called Allele Specific Locked Nucleic Acid quantitative PCR (ASLNAqPCR) based on 3′-locked nucleic acid (LNA)-modified primers and the use of a LNA-modified beacon probe. The assay is very cost-effective and not only identifies mutations with high specificity and sensitivity, but unlike other methods gives reliable information about the ratio of mutant and wild-type alleles. We have utilized ASLNAqPCR to identify the most common codon 12 and 13 *KRAS* mutations and the *BRAF* V600E mutation, but the test can be easily adapted to detect hot spot mutations in other oncogenes.

## Materials and Methods

### Ethics Statement

Since KRAS and BRAF mutational analysis is part of proper diagnostic protocols, the need for ethic committee's approval was not necessary for this study, in accordance with medical ethical guidelines of the Azienda Unita' Sanitaria Locale di Bologna (Ufficio Qualita' di Sistema Aziendale, Via Castiglione 29, 40100 Bologna). Accordingly to these guidelines, a comprehensive written informed consent was signed for the surgical treatment that produced the tissue samples and the related diagnostic procedures. All information regarding the human material used in this study was managed using anonymous numerical codes, clinical data were not used and samples were handled in compliance with the Helsinki declaration (http://www.wma.net/en/30publications/10policies/b3/).

### Selection of tumor material

Three hundred consecutive tumor samples from the Department of Pathology of the Azienda Unita' Sanitaria Locale di Bologna Ospedale Bellaria-Università di Bologna and corresponding to 281 patients, were analyzed. Of the 300 samples, 220 were primary tumours: 163 from the colon, 29 from the lung, 21 from the pancreas-9 adenocarcinomas and 12 cyst fluid aspirates from pancreatic neoplasms-and 7 from the thyroid. The remaining 80 samples were metastases at various sites from primary tumors of the colon (n = 71) or lung (n = 9) (Table 1 and Table 2). Two hundred and seventy-six samples were obtained from routinely processed formalin-fixed paraffin embedded (FFPE) sections (187 surgical specimens, 89 biopsy samples); 21 were fine needle cytology aspirates from pancreatic and 3 from lung lesions. For FFPE material, Hematoxylin and Eosin (H&E) sections were reviewed to identify paraffin blocks with the highest relative amount of tumor vs. stroma, few infiltrating lymphocytes and little or no tumor necrosis. Six 10 μm thick sections were cut from each block, followed by one H&E control slide. The tumor area selected for the analysis was marked on the control slide to ensure, whenever possible, greater than 70% content of neoplastic cells, in accordance with published guidelines [8]. Tumor material was manually dissected under microscopic guidance from the corresponding 10 μm sections using a sterile blade. Dissected tumor areas ranged from 0.25–1.0 cm^2^. For cytology preparations the slides with the highest tumor content were selected and material collected after removal of the coverslip. All patient information was handled in accordance with review board approved protocols and in compliance with the Helsinki declaration (http://www.wma.net/en/30publications/10policies/b3/).

**Table 1:** *KRAS* mutations in 300 consecutive samples analyzed by Sanger sequencing and ASLNAqPCR.

Tissue	*KRAS*		*No amplifiable DNA*	
	*Mutated by SSEQ (%)*	*Mutated by ASLNA (%)*	*SSEQ (%)*	*ASLNA (%)*
**COLON ** ***CRC (*** **n = 234)**	**80/209 (38.3)**	**94/221 (44.8)**	**25/234 (10.7)**	**13/234 (5.6)**
***Primary (n = 163)***	56/146 (38.4)	69/153 (45.1)	17/163 (10.4)	10/163 (6.1)
***Metastatic (n = 71)***	24/63 (38.1)	25/68 (36.7)	8/71 (11.3)	3/71 (4.2)
**LUNG ** ***NSCLC (*** **n = 38)**	**16/38 (42.1)**	**17/37 (45.9)**	**0/38 (0)**	**1/38 (0)**
***Primary (n = 29)***	9/29 (31.0)	12/29 (41.4)	0/29 (0)	0/29 (0)
***Metastatic (n = 9)***	7/9 (77.8)	5/8 (62.5)	0/9 (0)	1/9 (11.1)
**PANCREAS (n = 21)**	**7/21 (33.3)**	**6/21 (28.6)**	**0/21 (0)**	**0/21 (0)**
***Carcinoma (n = 9)***	4/9 (44.4)	4/9 (44.4)	0/9 (0)	0/9 (0)
***Cyst Fluid (n = 12)***	3/12 (25.0)	2/12 (16.7)	0/12 (0)	0/12 (0)
**THYROID (n = 7)**	**0/6 (0)**	**0/7 (0)**	**1/7 (14.3)**	**0/7 (0)**
***PTC-Classic (n = 3)***	0/3 (0)	0/3 (0)	0/3 (0)	0/3 (0)
***PTC-Others (n = 4)***	0/3 (0)	0/4 (0)	1/4 (25)	0/4 (0)

SSEQ, Sanger sequencing; ASLNA, allele specific quantitative PCR using 3′-locked nucleic acid modified primers (ASLNAqPCR); CRC, colonic adenocarcinoma; NSCLC, lung adenocarcinoma; PTC, papillary thyroid carcinoma.

**Table 2:** *BRAF* mutations analysis in 201 consecutive samples analyzed by Sanger sequencing and ASLNAqPCR.

Tissue	*BRAF*		*No amplifiable DNA*	
	*Mutated by SSEQ (%)*	*Mutated by ASLNA (%)*	*SSEQ (%)*	*ASLNA (%)*
**COLON ** ***CRC (*** **n = 159)**	**15/153 (9.8)**	**19/153 (12.4)**	**6/159 (3.8)**	**6/159 (3.8)**
***Primary (n = 114)***	13/109 (11.9)	15/109 (13.8)	5/114 (4.4)	5/114 (4.4)
***Metastatic (n = 45)***	2/44 (4.5)	4/44 (9.1)	1/45 (2.2)	1/45 (2.2)
**LUNG ** ***NSCLC*** ** (n = 24)**	**0/24 (0)**	**0/24 (0)**	**0/24 (0)**	**0/24 (0)**
***Primary (n = 17)***	0/17 (0)	0/17 (0)	0/17 (0)	0/7 (0)
***Metastatic (n = 7)***	0/7 (0)	0/7 (0)	0/7 (0)	0/7 (0)
**PANCREAS (n = 11)**	**0/10 (0)**	**0/10 (0)**	**1/11 (9.1)**	**1/11 (9.1)**
***Carcinoma (n = 5)***	0/5 (0)	0/5 (0)	0/5 (0)	0/5 (0)
***Cyst Fluid (n = 6)***	0/5 (0)	0/5 (0)	1/6 (16.7)	1/6 (16.7)
**THYROID (n = 7)**	**0/7 (0)**	**1/7 (1.4)**	**0/7 (0)**	**0/7 (0)**
***PTC-Classic (n = 3)***	0/3 (0)	1/3 (0)	0/3 (0)	0/3 (0)
***PTC-Others (n = 4)***	0/4 (0)	0/4 (0)	0/3 (0)	0/3 (0)

SSEQ, Sanger sequencing; ASLNA, allele specific quantitative PCR using 3′-locked nucleic acid modified primers (ASLNAqPCR); CRC, colonic adenocarcinoma; NSCLC, lung adenocarcinoma; PTC, papillary thyroid carcinoma.

### Cell line controls

The SW620 (*KRAS* G12V homozygous, ATCC – American Type Culture Collection, Rockville, MD, USA), CAL62 (*KRAS* G12R heterozygous), OCUT (*BRAF* V600E heterozygous), ARO (*BRAF* V600E heterozygous) and TPC-1 (*BRAF* wild type, American Type Culture Collection, Rockville, MD, USA) cell lines were used as DNA controls for mutational analysis. The CAL62, OCUT and ARO cell lines have been previously described [19] and were kindly provided by Prof. M. Santoro (University of Naples, Italy). Mutant DNA extracted from the cell lines was spiked in a pool of healthy female donor DNA (DNA Female pool, Cod. G1521, Promega, Madison WI) and serially diluted as 50%, 20%, 10%, 5%, 1%, 0,1%, 0.01% mutant to wild type DNA ratios to determine the analytical sensitivity of both Sanger sequencing and ASLNAqPCR. The minimal amount of input DNA required to obtain reliable mutation detection with the ASLNAqPCR method was determined by serially diluting DNA of the G12V mutated SW620 cell line with normal DNA, as previously described [20].

### Primers and molecular beacon probes design

**Figure 1:**
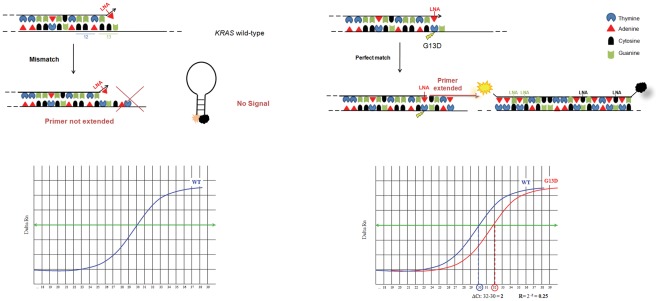
Diagram illustrating ASLNAqPCR. Left side: a single mismatch of the LNA modified primer does not allow PCR amplification. Right side: in case of a perfect match, the *Taq* polymerase extends the DNA strand and the amplicon is detected by the internal LNA modified beacon probe.

Primers and molecular beacon probes for ASLNAqPCR were designed using Primer3 software (http://frodo.wi.mit.edu/primer3/) (Table 3). They identify the seven most common *KRAS* mutations at codons 12 and 13, present in greater than 95% of tumors with mutated *KRAS* [3] and the *BRAF* V600E present in >90% of tumors with a *BRAF* mutation [9]. Forward ASLNAqPCR mutation-specific primers were modified with LNA nucleotides [21] at the 3′-end terminal of the oligonucleotide sequence (Table 3 and Figure 1). Two internal LNA-modified molecular beacon probes were designed, one for *KRAS* and one for *BRAF* real-time analysis (Table 3 and Figure 1). Flanked molecular beacon arms were designed using the OLIGO 6.0 software reaching a temperature between 57°C and 61°C in the stem loop conformation. All primers and probes were tested by *MFOLD* (http://www.bioinfo.rpi.edu/applications/mfold/old/dna/) to avoid secondary structures. Table 3 also shows the standard set of primers for *KRAS* [3] and *BRAF* [22] used for Sanger sequencing.

**Table 3:** Primers and beacon probes.

Sanger Sequencing			
Gene	Exon	Forward Primer	Reverse Primer
KRAS	2	AAGGTGAGTTTGTATTAAAAGGTACTGG	TGGTCCTGCACCAGTAATATGC
	3	TCCAGACTGTGTTTCTCCCTTCTC	AAAACTATAATTACTCCTTAATGTCAGCTT
BRAF	15	TCATAATGCTTGCTCTGATAGGA	GGCCAAAAATTTAATCAGTGGA

ASLNAqPCR			
Gene	WT/Mutation	Forward Primer	Reverse Primer
KRAS	WT	GGTAGTTGGAGCTGGTGGC	AGAGTGCCTTGACGATACA
	G12A	TGTGGTAGTTGGAGCTG+C	AGAGTGCCTTGACGATACA
	G12C	CTTGTGGTAGTTGGAGCT+T	AGAGTGCCTTGACGATACA
	G12D	GTGGTAGTTGGAGCTG+A	AGAGTGCCTTGACGATACA
	G12R	CTTGTGGTAGTTGGAGCT+C	AGAGTGCCTTGACGATACA
	G12S	TTGTGGTAGTTGGAGCT+A	AGAGTGCCTTGACGATACA
	G12V	TTGTGGTAGTTGGAGCTG+T	AGAGTGCCTTGACGATACA
	G13D	GTAGTTGGAGCTGGTG+A	AGAGTGCCTTGACGATACA
	BEACON	5′-FAM-CCGGTGAAGA+GT+GCCTTGA+CGATA+CAGCACCGG-BH1 -3′	
BRAF	WT	TAGGTGATTTTGGTCTAGCTACAG+T	TTAATCAGTGGAAAAATAGCCTCA
	V600E	TAGGTGATTTTGGTCTAGCTACAG+A	TTAATCAGTGGAAAAATAGCCTCA
	BEACON	5′-FAM-CCGAAGGGGATC+CAGACAA+CTGTTCAAACTGCCTTCGG-3BHQ-1 -3′	

bp, base pair. “+” precedes LNA-modified nucleotides.

### Mutational Analysis

DNA was extracted from FFPE using the RecoverAll kit (Ambion, Austin TX, U.S.A.), according to the manufacturer's recommendation. DNA from cell lines and FNA samples was extracted using the Gentra Puregene Kit (Qiagen, Hilden, Germany). DNA concentration was measured using the Quant-iT™ dsDNA BR kit (Invitrogen, Carlsbad, CA).

#### a) Sanger Sequencing

All 300 samples were tested for *KRAS*, 201 for *BRAF*. Exon 2 and 3 of *KRAS* and exon 15 of *BRAF* were evaluated amplifying fragments of 264 bp, 257 bp, and 223 bp respectively, similar to what previously described [3], [22]. PCR reactions were performed using the FastStartTaq DNA polymerase (Roche Applied Science, Mannheim, Germany) following the instructions of the provider, starting from 30–50 ng for DNA from FFPE and from about 15 ng for cell line DNA. The cycling conditions are shown in Table 4. Sequencing was carried out according to standard procedures using the GenomeLab DTCS Kit (Beckman Coulter, Inc., Fullerton, CA, U.S.A.) and a CEQ2000 XL automatic DNA sequencer (Beckman Coulter, Inc., Fullerton, CA, U.S.A). Strands were screened using forward and reverse primers.

**Table 4:** PCR conditions for Sanger sequencing and ASLNAqPCR.

Sanger Sequencing			
*Amplicon*	*Temperature*	*Time*	*Cycles*
*BRAF* (Ex15)	95°C	4′	1
	95°C	30″	40
	53°C	30″	40
	72°C	30″	40
	72°C	10′	1
*KRAS* (Ex2 and Ex3)^a^	95°C	4′	1
	95°C	30′	5
	63°C–1°C/cycle	30′	5
	72°C	30″	5
	95°C	30″	35
	58°C	30″	35
	72°C	30″	35
	72°C	10′	1

ASLNAqPCR			
*Amplicon*	*Temperature*	*Time*	*Cycles*
*KRAS* ^b^ *BRAF* ^c^	50°C	2′	1
	95°C	10′	1
	95°C	30″	38
	60°C*	30″	38
	72°C	30″	38

Ex, exon; ^a^For *KRAS* amplification a touch-down PCR was performed; ^b^PCR for wild type *KRAS* and all 7 codon 12 and 13 mutations;^ c^PCR for wild type *BRAF* and *BRAF* V600E; *Plate reading step.

#### b) ASLNAqPCR

All 300 samples were tested for *KRAS*, 201 for *BRAF*. Fifteen nanograms of DNA purified from fresh cell lines, or 15–50 ng of DNA purified from FFPE, were amplified using the FastStart Universal Probe Master with ROX (Roche Applied Science, Mannheim, Germany) in separate real time reactions for each allele specific primer, but in the same run and following the same cycling conditions shown in Table 4. PCR products were 117 bp for *BRAF* V600E and 104 to 110 bp for *KRAS*. Real-time PCR was performed using an ABI SDS 7000TM instrument (Applied Biosystems, Foster City, CA). The relative mutant allele copy number was quantified during the exponential phase of real-time PCR using the ΔCT method [23]. Samples with quantification cycles above 35 for the wild type allele were considered failures and excluded from the study.

#### c) Pyrosequencing

Twenty-one samples with discrepant results between Sanger sequencing and ASLNAqPCR were tested by pyrosequencing, according to standard procedures using PyroMark Gold Q96 (Qiagen, Gmbh, Hilden Germany) reagents and a PyroMarkTM Q96 ID instrument. Pyrograms outputs were analysed with PyroMarkTM Q96 ID Software (Qiagen, Gmbh, Hilden Germany) using the allele quantification (AQ) mode.

#### Statistical measures of performance

True positive (TP), false positive (FP), true negative (TN), false negative (FN), test sensitivity (SEN), specificity (SPEC), negative predictive value (NPV), positive predictive value (PPV), accuracy (ACC), false discovery rate (FDR) [24].

## Results

Of the 300 consecutive cases, 201 were analyzed for both *KRAS* and *BRAF* and 99 only for *KRAS*. Ten of 300 cases analyzed for *KRAS* gave no amplifiable products due to excessive DNA degradation with both Sanger sequencing and the ASLNAqPCR technique. Sixteen additional cases gave amplifiable *KRAS* PCR products by ASLNAqPCR but not by sequencing and four additional cases by sequencing but not by ASLNAqPCR. Seven of 201 cases analyzed for *BRAF* gave no amplifiable products due to excessive DNA degradation with both Sanger sequencing and ASLNAqPCR.

### ASLNAqPCR analytical sensitivity, intra– and inter-assay reproducibility, failure rate

#### Analytical sensitivity-Sanger sequencing

Analytical sensitivity was tested by serially diluting DNA from the G12V mutated SW620 cell line, the G12R mutated CAL62 cell line, the *BRAF* V600E mutated ARO cell line, the *BRAF* V600E mutated OCUT cell line in a pool of healthy female donor DNA. The TPC-1 cell line was used as non-mutated control for the dilution tests. At least 20% of *KRAS* G12V and *KRAS* G12R DNA were required to identify the mutations. The *BRAF* V600E mutation was identified with at least 10% of mutated DNA.

#### Analytical sensitivity-ASLNAqPCR

**Figure 2:**
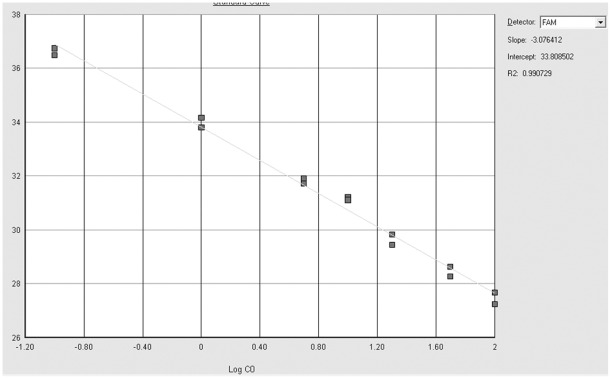
Standard curve titration of ASLNAqPCR for *KRAS*. Serial dilution of the *KRAS G12V* mutated SW620 cell line DNA in wild type DNA. Gray squares correspond to 50%, 20%, 10%, 5%, 1%, 0,1%, 0.01% mutant to wild type DNA ratios (duplicate samples). The titration slope is −3.076, R^2^ is 0.991 (top right), corresponding to a PCR efficiency of 111.3%.

**Figure 3:**
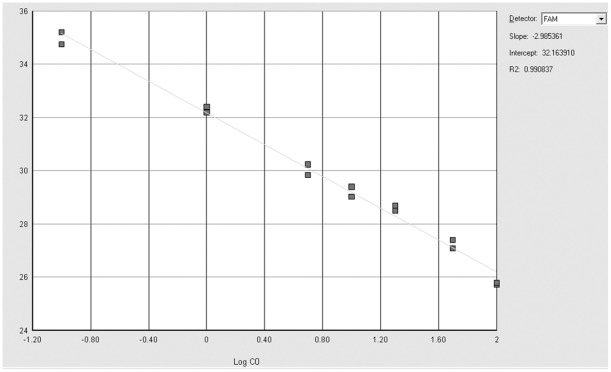
Standard curve titration of ASLNAqPCR for *BRAF.* Serial dilution of the *BRAF V600E* mutated OCUT cell line DNA in wild type DNA. Gray squares correspond to 50%, 20%, 10%, 5%, 1%, 0,1%, 0.01% mutant to wild type DNA ratios (duplicate samples). The titration slope is −2.985, R^2^ is 0.991 (top right), corresponding to a PCR efficiency of 116.2%.

Analytical sensitivity was tested with the same mutated DNA dilutions used for the Sanger sequencing. The *KRAS* G12V and *KRAS* G12R mutations were reproducibly detectable at a dilution of 0.1% with a PCR efficiency of 111.3% (slope: −3.0764, R^2^: 0.9907) [23] (Figure 2). The *BRAF* V600E mutation was reproducibly detected at a dilution of 0.1% with a PCR efficiency of 116.2% (slope: −2.9854, R^2^: 0.9908) [23] (Figure 3).

#### Minimal amount of input DNA for ASLNAqPCR at the analytical sensitivity threshold

The amount of a 0.1% dilution of *KRAS* G12V mutated SW620 cell line DNA and of *BRAF* V600E mutated ARO cell line DNA spiked with normal DNA was serially decreased to determine the minimal input DNA necessary for mutation detection. A minimal amount of 6.25 ng of DNA from cell lines (equivalent to ∼1000 copies of a diploid human genome) was necessary to detect both mutations. ASLNAqPCR analysis of all clinical samples below the 6.25 ng input DNA threshold was therefore repeated starting with a higher amount of tumor tissue.

#### ASLNAqPCR intra– and inter-assay reproducibility

Intra-assay reproducibility (i.e. the consistency of results in the same run) has been measured by calculating the Ct (cycle threshold) coefficients of variation of samples run as duplicate in the same plate using serial dilution (50%, 20%, 10%, 5%, 1%, 0,1%) of mutant DNA in wild type DNA for *KRAS* (CAL62 and SW620 cell lines) and *BRAF* (ARO and OCUT cell lines). The coefficients of variation for the *KRAS* mutated DNA ranged between 0.08% and 1.03%. Those for the *BRAF* mutated DNA ranged between 0.15% and 1.54%. Inter-assay reproducibility (i.e. the consistency of results with the same protocol but in different runs) has been similarly measured by calculating the Ct coefficient of variation of duplicate samples run in different days using the same serial dilutions of mutated DNA mentioned above. The coefficients of variation for the *KRAS* mutated DNA ranged between 0.91% and 1.62%. Those for the *BRAF* mutated DNA ranged between 1.12% and 1.73%. Both intra– and inter assay reproducibility results are well within the 10% range considered satisfactory [25].

#### ASLNAqPCR failure rate

The failure rate was tested by repeating a series of samples with a mutated/wild type ratio thrice the analytical sensitivity threshold, according to published guidelines [26]. Twenty-four samples with a 0.3% dilution of *KRAS* G12V SW620 cell line DNA and of *BRAF* V600E ARO cell line DNA were tested. The failure rate was zero, as mutations were consistently detected in all cases.

### KRAS and BRAF mutation analysis

#### Sanger sequencing analysis

Two-hundred-seventy-four samples gave amplifiable DNA and 103 of them (37.6%) showed a *KRAS* mutation at codon 12, 13 or 61 (Table 1 and Table 5). Out of these, five cases had mutations in exon 3 codon 61 (Q61H or Q61L), not detectable by our *ASLNAqPCR* method. Fifteen out of the 194 evaluable cases (7.7%) showed the *BRAF* V600E mutation (Table 2 and Table 5). No *BRAF* exon 15 mutations other than the V600E were detected. *KRAS* and *BRAF* mutations were mutually exclusive on all cases.

**Table 5:** Frequence of specific *KRAS* and *BRAF* mutations cases analyzed by SSEQ and ASLNAqPCR.

Gene	Mutation	SSEQ (%)	ASLNA (%)
*KRAS (n = 300)*	G12D	41/274 (14.9)	48/286 (16.8)
	G12V	23/274 (8.4)	26/286 (9.1)
	G13D	17/274 (6.2)	19/286 (6.6)
	G12C	8/274 (2.9)	12/286 (4.2)
	G12S	3/274 (1.1)	4/286 (1.4)
	G12A	3/274 (1.1)	3/286 (1.1)
	G12R	2/274 (0.7)	5/286 (1.7)
	G12F	1/274 (0.4)	NT
	Q61H	3/274 (1.1)	NT
	Q61L	2/274 (0.7)	NT
	All mutant cases	103/274 (37.6)	117/286 (40.9)
	No amplifiable DNA	26/300 (8.7)	14/300 (4.7)
*BRAF (n = 201)*	V600E	15/194 (7.7)	20/194 (10.3)
	No amplifiable DNA	7/201 (3.5)	7/201 (3.5)

SSEQ, Sanger sequencing; ASLNA, allele specific quantitative PCR using 3′-locked nucleic acid modified primers (ASLNAqPCR); NT, not tested, since ASLNAqPCR primers were designed to identify only the seven most common codon 12−13 *KRAS* mutations.

#### ASLNAqPCR analysis

Two-hundred-eighty-six samples gave amplifiable DNA and 117 of them (40.9%) showed a *KRAS* mutation at codon 12, 13 (Table 1 and Table 5), codon 61 mutations were not tested by *ASLNAqPCR*. Twenty of the 194 evaluable cases (10.3%) showed the *BRAF* V600E mutation (Table 2 and Table 5). No *BRAF* exon 15 mutations other than the V600E were tested by *ASLNAqPCR*. Quantitative real time data always indicated a mutant/wild type ratio equal to or less than 1, consistent with heterozygous mutations. As in the case of Sanger sequencing results, *KRAS* and *BRAF* mutations were always mutually exclusive.

The *KRAS* and *BRAF* V600E mutation rates detected in our series by both Sanger sequencing and ASLNAqPCR are compatible with the data reported in the literature for colon adenocarcinoma (Table 6) and the other tumors analyzed [3], [4], [5], [11], [16], [27].

**Table 6:** *KRAS* mutations in primary colon carcinoma (n = 163) compared with data reported in the literature

Mutation	SSEQ (%)	ASLNA (%)	Literature data, % values^a^
G12D	22/146 (15.1)	27/153 (17.7)	12.9–15.5
G12V	14/146 (9.6)	15/153 (9.8)	7.7–12.2
G13D	8/146 (5.5)	10/153 (6.5)	5–7.3
G12C	5/146 (3.4)	7/153 (4.4)	2.3−3.6
G12A	3/146 (2.1)	3/153 (2.0)	2.3–2.8
G12S	3/146 (2.1)	4/153 (2.6)	2.6–4.3
G12R	0/146 (0)	3/153 (2.0)	0.3–0.5
G12F	1/146 (0.7)	NT	0.2
Q61H	0/146 (/)	NT	0.1
Q61L	0/146 (/)	NT	0.1
All mutant cases	56/146 (38.4)	69/153 (45.1)	37–42.6
No amplifiable DNA	17/163 (10.4)	10/163 (6.1)	___

ASLNA, allele specific quantitative PCR using 3′-locked nucleic acid modified primers (ASLNAqPCR); NT, not tested, since ASLNAqPCR primers were designed to identify only the seven most common codon 12–13 *KRAS* mutations. ^a^ References [Bamford et al., 2004; Karapetis et al., 2008; Neumann et al., 2009].

#### Comparison between Sanger and ASLNAqPCR and pyrosequencing of samples with discordant results

**Figure 4:**
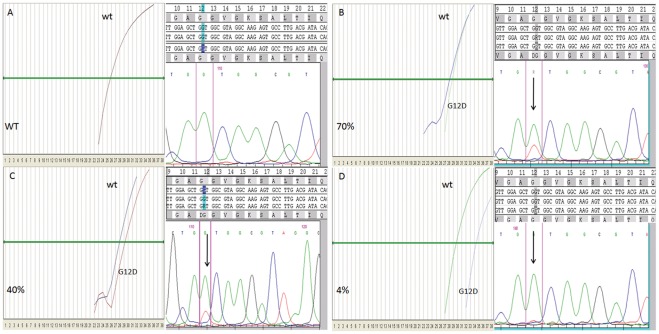
ASLNAqPCR and corresponding Sanger sequencing of four representative tumor samples analyzed for *KRAS* mutations. Sample A is wild type, samples B, C and D are *KRAS G12D* mutated with varying amounts of tumor vs. non neoplastic cells; assuming that *KRAS G12D* is heterozygous, quantitation of mutated DNA by ASLNAqPCR (ΔCT method) is consistent with 70% of mutated cells in sample B, 40% of mutated cells in sample C, 4% of mutated cells in sample D; in sample D the *KRAS G12D* mutation is detected only by the ASLNAQPCR due to its high analytical sensitivity.

**Figure 5:**
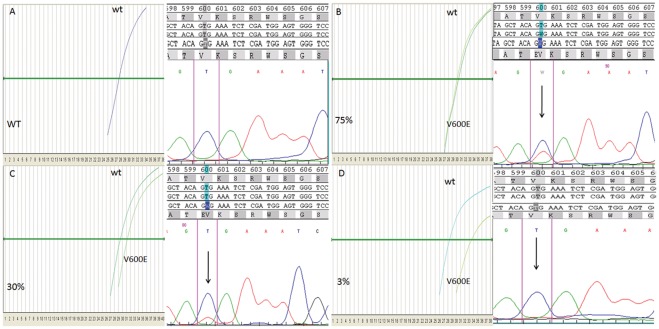
ASLNAqPCR and corresponding Sanger sequencing of four representative tumor samples analyzed for the *BRAF V600E* mutation. Sample A is wild type, samples B, C and D are *BRAF V600E* mutated with varying amounts of tumor vs. non neoplastic cells; assuming that *BRAF V600E* is heterozygous, quantitation of mutated DNA by ASLNAqPCR (ΔCT method) is consistent with 75% of mutated cells in sample B, 30% of mutated cells in sample C, 3% of mutated cells in sample D; in sample D the *BRAF V600E* mutation is detected only by the ASLNAQPCR due to its high analytical sensitivity.

**Figure 6:**
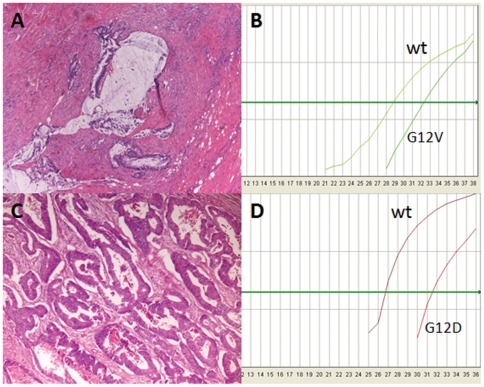
*KRAS* mutations identified by ASLNAqPCR but not by Sanger sequencing. A , Hematoxylin and Eosin (H&E) stained section (X100) of the area of case 13 of Table 7 (rectal adenocarcinoma treated with preoperative chemo– and radiation therapy) dissected for DNA extraction with a tumor vs. non neoplastic cell ratio of ∼10%, below the analytical sensitivity threshold of Sanger sequencing. **B**, ASLNAqPCR of case 13 of Table 7 shows a *KRAS* G12V mutation with a mutated/wild type ratio of 4%, corresponding to 8% mutated cells, assuming that the mutation is heterozygous; this is consistent with the mutation being present in the large majority of neoplastic cells. **C**, H&E stained section (X100) of the area of the colonic adenocarcinoma case 1 of Table 7, dissected for DNA extraction with a tumor vs. non neoplastic cell ratio of ∼70%. **D**, ASLNAqPCR of case 1 of Table 7 shows a *KRAS* G12D mutation with a mutated/wild type ratio of 1.5%, corresponding to 3% mutated cells, assuming that the mutation is heterozygous; this is consistent with a small *KRAS* G12D mutated subclone corresponding to ∼4% of the neoplastic cells.

Sanger sequencing and the ASLNAqPCR assay generated discordant results in 22/300 samples for *KRAS* mutations (7.3%) (Table 7) and in 5/201 samples for *BRAF* V600E (2.5%) (Table 8). Eighteen discordant *KRAS* samples and 3 discordant *BRAF* ones were further analyzed by pyrosequencing. No additional material was available to repeat the test in 4 *KRAS* and 2 *BRAF* mutated cases. Among the samples re-tested by pyrosequencing there were two mutated for *KRAS* Q61H and one for *KRAS* G12F, not detectable by ASLNAqPCR. Pyrosequencing confirmed all *KRAS* mutations identified by ASLNAqPCR but not detected by Sanger sequencing (Table 7, Figure 4). Importantly, quantitative real time data showed a mutated/wild type ratio ≤10% –below our *KRAS* Sanger sequencing analytical sensitivity threshold-for all these samples (Table 7). The same was true for *BRAF* real time data (Table 8, Figure 5). Review of the pathology material showed variable ratios of tumor to non-neoplastic cells in the areas that were manually dissected for mutational analysis where a mutation was detected by ASLNAqPCR, confirmed by pyrosequencing but not identified by Sanger sequencing. In the majority of samples the discrepant result was simply explained by the low tumor to non-neoplastic cell ratio and the higher analytical sensitivity of ASLNAqPCR (Figure 6, panels A and B). However, in some samples tumor heterogeneity was a contributing factor. ASLNAqPCR quantification of the mutated to wild type allele ratio clearly indicated the presence of tumor cell subclones in 7 of the 16 discrepant *KRAS* results (cases 1, 8, 9, 10, 11, 12, 16 of Table 7, Figure 6, panels C and D) and in 3 of the 5 discrepant *BRAF* results (cases 2, 3, 4 of Table 8). In these cases, ASLNAqPCR results and review of the tumor to non-neoplastic cell ratio in the area dissected for DNA extraction were consistent with mutated cells representing <30% of the tumor cell population, assuming that all mutations were heterozygous. In two cases data indicated that mutated cells represented <5% of the tumor (case 1 of Table 7 and case 2 of Table 8, Figure 6, panels C and D).

**Table 7:** *KRAS* Pyrosequencing analysis of cases with discordant results between ASLNAqPCR and Sanger sequencing.

Case number	KRAS-SSEQ	KRAS-ASLNA^a^	*KRAS* mutated/wild type (%)^b^	*KRAS* Pyrosequencing	Sample	Tumor cells/Non neoplastic cells (%)^d^
1	WT	G12D	1.5	G12D	CRC, resection	70
2	WT	G12C	3.0	G12C	NSCLC, biopsy	10
3	WT	G12D	8.0	G12D	CRC, resection	35
4	WT	G12R	4.0	G12R	CRC, resection	15
5	WT	G12R	4.0	G12R	CRC, resection	25
6	WT	G12S	8.0	G12S	CRC, resection	30
7	WT	G12C	7.0	G12C	CRC, resection	45
8	WT	G12V	5.0	G12V	CRC, resection	70
9	WT	G13D	7.0	G13D	CRC, resection	70
10	WT	G12D	6.0	G12D	CRC, resection	45
11	WT	G12D	2.0	G12D	NSCLC, biopsy	30
12	WT	G13D	3.0	G13D	CRC, resection	35
13	WT	G12V	4.0	G12V	CRC, resection^c^	10
14	WT	G12D	1.0	G12D	PC, FNA	<5
15	WT	G12C	3.0	G12C	NSCLC, biopsy	5
16	WT	G12V	10.0	NP	CRC, resection	80
17	G12F	G12C	20.0	G12F	CRC, resection	45
18	Q61H	WT	/	Q61H	metNSCLC, LN biopsy	50
19	Q61H	WT	/	Q61H	PC, FNA	60
20	Q61H	WT	/	NP	metCRC, liver biopsy	70
21	Q61L	WT	/	NP	metCRC, lung biopsy	50
22	Q61L	WT	/	NP	PC, FNA	40

SSEQ, Sanger sequencing; ASLNA, allele specific quantitative PCR using 3′-locked nucleic acid modified primers (ASLNAqPCR); WT, wild type; NP, not performed due to lack of additional DNA; CRC, colonic adenocarcinoma; NSCLC, lung adenocarcinoma; met, metastatic; LN, lymph node; FNA, fine needle aspirate; PC, pancreatic carcinoma. ^a^ASLNAqPCR primers designed to identify only the seven most common codon 12–13 *KRAS* mutations, codon 61 *KRAS* and G12F mutations are not detectable. ^b^Real time ASLNAqPCR quantitative data. ^c^Status post neoadjuvant chemo– and radiation therapy.^ d^Percentage of the tumor/non neoplastic cells ratio estimated in the area dissected for DNA extraction.

**Table 8:** *BRAF* Pyrosequencing analysis of cases with discordant results between ASLNAqPCR and Sanger sequencing.

Case number	*BRAF*-SSEQ	*BRAF*-ASLNA^a^	*BRAF* mutated/wild type (%)^b^	Pyrosequencing	Sample	Tumor cells/Non neoplastic cells (%)^c^
**1**	WT	V600E	1.25	V600E	metCRC, LN biopsy	5
**2**	WT	V600E	1.25	V600E	CRC, resection,	75
**3**	WT	V600E	1.5	V600E	CRC, resection	55
**4**	WT	V600E	3.0	NP	PTC, resection	80
**5**	WT	V600E	3.0	NP	CRC, resection	10

SSEQ, Sanger sequencing; ASLNA, allele specific quantitative PCR using 3′-locked nucleic acid modified primers (ASLNAqPCR); WT, wild type; NP, not performed due to lack of additional DNA; CRC, colonic adenocarcinoma; met, metastatic; LN, lymph node; PTC papillary thyroid carcinoma. ^a^ASLNAqPCR primers designed to identify only the *BRAF* V600E mutation. ^b^Real time ASLNAqPCR quantitative data. ^c^Percentage of the tumor/non neoplastic cells ratio estimated in the area dissected for DNA extraction.

The only cases mutated by Sanger sequencing and pyrosequencing for which a mutation was not identified by ASLNAqPCR were two cases with *KRAS* Q61H (cases 18 and 19 of Table 7), not tested by our ASLNAqPCR primers. In one additional case with a G12F (case 17 of Table 7) the Glycine to Phenylalanine mutation was due to a double nucleotide substitution from GGT to TTT on the same *KRAS* allele, as confirmed by pyrosequencing. The case was identified as mutated by the ASLNAqPCR primer specific for the Glycine to Cysteine mutation (G12C). This is due to the fact that the G12C specific primer correctly recognized the mutated Thymine at the first nucleotide position of the codon. The primer specific for G12V, that recognizes mutated thymines at the second position, was not able to anneal due to the presence, instead of the wild type Guanine, of the mutated Thymine at the beginning of the codon.

### Statistical measures of performance

Test sensitivity, specificity and other statistical measures of performance for ASLNAqPCR and Sanger sequencing are shown in Table 9. We considered a result true positive (TP), false positive (FP), true negative (TN) or false negative (FN) as follows. TP were cases that showed the same mutation by both Sanger sequencing *and* ASLNAqPCR; for cases that gave a discrepant results by the two methods, we considered true positives those with the mutation confirmed by pyrosequencing. FP were cases where a mutation found by one of the two methods (Sanger *or* ASLNAqPCR) was not confirmed by pyrosequencing. TN were cases that resulted wild type by both Sanger sequencing *and* ASLNAqPCR; for cases that gave a discrepant results by the two methods, we considered true negatives those that resulted wild type by pyrosequencing. FN were cases where a wild type result by one of the two methods (Sanger *or* ASLNAqPCR) resulted mutated by pyrosequencing. Test sensitivity (SEN), specificity (SPEC), negative predictive value (NPV), positive predictive value (PPV), accuracy (ACC) and false discovery rate (FDR) were calculated as follows: SEN  =  TP/(TP+FN); SPEC  =  TN/(TN+FP) ×100; NPV  =  TN/(TN+FN) ×100; ACC  =  (TP+TN)/(TP+FP+TN+FN) ×100; FDR  =  FP/(FP+VP). Our ASLNAqPCR was designed to detect the seven most common codon 12–13 *KRAS* mutations and did not detect codon 61 *KRAS* mutations. For statistical evaluation, all mutations not detected by ASLNAqPCR, including those for which allele specific primers were not designed, were scored as ASLNAqPCR “wild type” results. As shown in Table 9, ASLNAqPCR had 100% specificity, as did Sanger sequencing. The sensitivity of ASLNAqPCR was 95.19%, higher than that of Sanger sequencing (81.37%). Also accuracy and negative predictive value were greater for ASLNAqPCR compared with Sanger sequencing.

**Table 9:** Statistical measures of performance for Sanger sequencing and ASLNAqPCR.

Diagnostic Test	Specificity	Sensitivity	PPV	NPV	ACC	FDR
**SSEQ**	100%	81.4%	100%	90.0%	94.2%	0
**ASLNA**	100%	95.2%	100%	97.0%	98.2%	0

SSEQ, Sanger sequencing; ASLNA, allele specific quantitative PCR using 3′-locked nucleic acid modified primers (ASLNAqPCR); PPV, positive predictive value; NPV, negative predictive value; ACC, accuracy; FDR, false discovery rate.

## Discussion

The therapeutic use of tyrosine kinase receptors inhibitors (TKIs), like Cetuximab or Panitumumab for CRC and Gefitinib or Erlotinib for NSCLC that target EGFR, requires testing of the molecular effectors downstream to the membrane-bound tyrosine kinases and wild type status for these effectors is expected for response to TKIs therapy. Among these, *KRAS* and *BRAF* are commonly mutated and the absence of *KRAS* activating mutations is now a necessary condition to treat CRC patients with Cetuximab or Panitumumab. The need to screen for mutations in a large number of patient samples with rapid turnaround time is a strong motivation to develop methods that are cost-effective, reliable and robust.

Our ASLNAqPCR is a novel allele specific assay with forward mutation-specific primers modified with LNA nucleotides at the 3′-end sequence terminal and an internal LNA-modified beacon probe that detects and quantifies oncogenic mutations with high specificity and sensitivity. Allele specific PCR is ideally suited to detect oncogenic mutations when these are caused by relatively few nucleotide changes at specific hot spots of the gene. However, natural DNA primers in conventional allele specific PCR can miss-anneal the target sequence, particularly when PCR conditions are suboptimal (e.g. due to DNA damaged by formalin fixation or degraded, limiting amounts of the target sequence), thus causing false positive results that may have unwanted consequences for TKI patient treatment.

LNAs are nucleic acid analogs with a 2′-O, 4′-C methylene bridge that “locks” the ribose into a C3′-endo conformation. When LNA-modified nucleotides are incorporated in oligonucleotides the melting DNA heteroduplex temperature (T_m_) increases between 1–8°C per LNA-modified nucleotide [21]. Because of the increased T_m_, LNA-modified nucleotides have been used for a variety of applications, including in situ hybridization [28], whole genome amplification [29], methylation sensitive PCR [20], germline SNP genotyping [21], as blocker oligonucleotides to suppress wild-type alleles and increase PCR sensitivity. Blocker LNA oligonucleotides have been shown to be particularly useful to detect oncogene mutations with high sensitivity, including *KRAS* and *BRAF* [30]. We tested allele specific primers made of unmodified DNA, but with the same base sequence shown in Table 3 for KRAS and BRAF, observing a consistent reduction in PCR specificity compared with the corresponding LNA-modified primers. When performing Allele Specific PCR without LNA modified primers we had false positive results in non-neoplastic samples, including DNA extracted from peripheral blood leukocytes. Specifically, four DNA samples from healthy blood donors and a pool of healthy female donor DNA tested with Allele Specific PCR showed bands compatible with KRAS and BRAF mutations on the agarose gel. The same samples were wild type when tested using ASLNAqPCR with LNA modified primers and probe and after Sanger sequencing (data not shown). In fact, LNA modification has been shown to greatly enhance allelic specificity, while maintaining a high level of sensitivity in comparison with conventional unmodified, natural DNA primers [21].

We have validated ASLNAqPCR analyzing 300 consecutive samples of routinely processed CRC, NSCLC, pancreatic and thyroid tumors, including both primary and metastatic lesions, surgical specimens, biopsy samples and cytology preparations. ASLNAqPCR identified *KRAS* and *BRAF* mutations with rates comparable to those reported in the literature. The test was “robust” with excellent intra– and inter-assay reproducibility and with only few routine samples that gave no amplifiable PCR. There were no ASLNAqPCR failures after repeated testing of a limiting ratio of *KRAS* and *BRAF* mutated cell line DNA/wild type DNA. ASLNAqPCR was performed in parallel with conventional Sanger sequencing on all cases, results were compared, and discrepant cases analyzed by pyrosequencing to statistically measure the performance of the assay. Our data demonstrate that ASLNAqPCR has 100% specificity and positive predictive value, with higher sensitivity, negative predictive value and accuracy compared with Sanger sequencing. We observed no false positive results, although if qPCR conditions are pushed above 40 cycles false positive results may be expected [21].

The only mutated samples identified by Sanger sequencing and confirmed by pyrosequencying, but not detected by ASLNAqPCR, were two *KRAS* Q61H mutations. One additional case had a rare double nucleotide substitution at *KRAS* codon 12 that ASLNAqPCR recognized as mutated but with the wrong aminoacid call. None of these could be identified because our allele specific primers were not designed to cover all possible codon 12 and 13 *KRAS* mutations, but only the most frequent, and we did not design primers for codon 61 *KRAS* mutations. A limitation of ASLNAqPCR, common to all hot spot mutation assays, is that it identifies – by definition – only the targeted mutation, while Sanger sequencing can identify all mutations present in the PCR amplicon. Had our study been limited to codon 12 and 13 *KRAS* mutations, ASLNAqPCR would have been ∼100% sensitive. The way ASLNAqPCR is designed allows for the easy addition of other RAS allele specific primers. In fact, the test can be conveniently adapted to identify hot spot mutations in other genes and we have successfully utilized ASLNAqPCR to identify IDH1-R132H mutation with high specificity and sensitivity in a series of more than 100 gliomas (data not shown).

In all cases where *KRAS* or *BRAF* mutations were detected by ASLNAqPCR, but not by Sanger sequencing, this was due to the higher analytical sensitivity of the assay. ASLNAqPCR assay can identify point mutations against a large excess of wild-type allele, in the thousand-fold range. We detected 0.1% *KRAS* and 0.1% *BRAF* mutated cell line DNA with high PCR efficiency, even when DNA for mutational analysis was as little as 6.25 ng, the minimal amount of input DNA at the analytical sensitivity threshold of the method. The ASLNAqPCR analytical sensitivity is thus much higher than that of conventional Sanger sequencing (∼25% mutated DNA) and higher than that reported with very sensitive methods such as pyrosequencing (1.25–2.5% mutated DNA) or ARMS PCR with scorpion oligonucleotides (TheraScreen) (1.25% mutated DNA) [31]. ASLNAqPCR is therefore ideally suited to confidently detect mutations in samples were the abundance of inflammatory cells, fibroblasts lymphocytes or other stromal elements results in tumor to non-neoplastic cell ratios that are below the recommended Sanger sequencing threshold of 50% tumor cells – corresponding to 25% DNA with heterozygous mutations [7]. In many of these samples the proportion of tumor cells cannot be effectively enriched by manual dissection. This is a common occurrence when the only material available for testing are metastatic deposits in lymph nodes, samples from patients that have undergone preoperatory chemo– and radiation treatment and fine needle aspiration specimens [30]. On the other hand, when tumor cells are abundant, the use of ASLNAqPCR makes the dissection of tumor material unnecessary, thus obviating the need for a laborious step that is time consuming and increases the potential for sample contamination.

One relevant feature of ASLNAqPCR compared with tests currently utilized to detect oncogenic mutations is that it allows for precise quantification of the mutated allele due to the LNA-modified beacon probe used for real time analysis. This is important when a method with high analytical sensitivity is utilized, since comparison of quantitative mutational data with the proportion of tumor cells in the samples analyzed allows to easily discriminate those where the mutation is widespread from those where the mutation is present only in small neoplastic cell clones. This was clearly the case in a few of our specimens, including both colorectal and lung adenocarcinomas, where quantitative ASLNAqPCR results of *KRAS* and *BRAF* analysis showed that mutations were present in a minority of the tumor cells. Although our data do not indicate that this is a particularly common occurrence, the presence of mutated subclones can be an issue for individual cases. Had the DNA from these patients been analyzed with a method that has high analytical sensitivity (e.g. pyrosequencing), but that does not allow precise quantification of the mutated allele, the tumors would have been diagnosed as mutated. This can, at least in the case of *KRAS*, deny a potentially beneficial treatment to CRC patients whose response to TKI may not be affected by the presence of small mutated clones. On the other hand, Sanger sequencing would have scored the case as negative and failed to predict a possible limited response to TKI. Since tumors are not always made of homogeneous cell populations, their heterogeneity is a relevant concern for therapies that have specific molecular targets. It is currently unclear if and how the presence of small clones with DNA mutations affects the clinical response to molecular inhibitors of oncogenic pathways [32], [33]. The presence and successive selection of mutated clones may indeed explain response failures in some patients [34]. Although the impact of tumor heterogeneity in deciding patient management is a matter of debate, quantitative mutational data may help to clarify the issue, while providing the oncologist with accurate data to manage the patient.

In addition to quantifying the mutation, ASLNAqPCR has considerable practical advantages over other currently used methods. The assay can be performed in any laboratory with real-time PCR equipment, LNA-modified primers and probes can be easily obtained at low cost and no proprietary reagents, other than those for TaqMan chemistry, are necessary. Once DNA has been extracted, few steps are required for the analysis. All reactions, seven for the *KRAS* mutations and one for *KRAS* wild type, one for *BRAF* V600E and one for *BRAF* wild type, have been optimized for a single real time run with identical cycling conditions. The entire procedure can be completed in ∼3 hours, including ∼30 minutes operator time to load a 96 well plate, ∼1 hour and 30′ for the real-time run and ∼10′ for data analysis. The short time for the analysis makes it possible to perform several runs in the same day. In addition, since samples are analysed in real-time there is no post-PCR manipulation, avoiding any risk of carry-over contamination.

In summary, we report and validate ASLNAqPCR. The test is rapid, cost-effective, highly sensitive and can accurately quantify oncogenic mutations. It can be proposed as a method of choice to analyze those samples that can not be enriched in neoplastic cell content by tumor dissection prior to DNA extraction. We validated the assay with primers designed to detect the most common *KRAS* and *BRAF* mutations in routinely processed samples, but ASLNAqPCR can easily be adapted to detect hot spot mutations in other oncogenes.
